# Life Satisfaction and Risk of Chronic Diseases in the European Prospective Investigation into Cancer and Nutrition (EPIC)-Germany Study

**DOI:** 10.1371/journal.pone.0073462

**Published:** 2013-08-20

**Authors:** Silke Feller, Birgit Teucher, Rudolf Kaaks, Heiner Boeing, Matthaeus Vigl

**Affiliations:** 1 Department of Epidemiology, German Institute of Human Nutrition Potsdam-Rehbruecke, Nuthetal, Germany; 2 Division of Cancer Epidemiology, German Cancer Research Center, Heidelberg, Germany; CUNY, United States of America

## Abstract

**Objective:**

The aim of the study was to examine the prospective association between life satisfaction and risk of type 2 diabetes mellitus, myocardial infarction, stroke, and cancer. Previous studies suggested that psychosocial factors may affect the development of chronic diseases but the impact of positive attitudes, in particular life satisfaction, is yet to be determined.

**Methods:**

The analysis included 50,358 participants of the European Prospective Investigation into Cancer and Nutrition (EPIC)-Germany study in Potsdam and Heidelberg. Life satisfaction was assessed in a baseline interview and incident cases of chronic diseases were identified and verified during follow-up. Hazard ratios were calculated using Cox proportional hazards regression models that were systematically multivariable-adjusted for established risk factors and prevalent diseases.

**Results:**

During an average of 8 years of follow-up 2,293 cases of cancer, 1,840 cases of type 2 diabetes mellitus, 440 cases of stroke, and 562 cases of myocardial infarction were observed. Women who were unsatisfied with life at baseline showed in all models a significantly increased risk of cancer (HR: 1.45; 95% CI: 1.18-1.78) and stroke (HR: 1.69; 95% CI: 1.05-2.73) as well as an increased risk of type 2 diabetes mellitus by trend across categories (p-trend=0.04) compared to women very satisfied with life. In men, a relationship between life satisfaction and stroke was found but did not persist after consideration of lifestyle factors and prevalent diseases. No significant association was observed between life satisfaction and risk of myocardial infarction.

**Conclusions:**

The results of this study suggest that reduced life satisfaction is related to the development of chronic diseases—particularly in women and partly mediated by established risk factors.

## Introduction

In 1946 the World Health Organization (WHO) defined health as ”a state of complete physical, mental and social well-being and not merely the absence of disease or infirmity” [1]. Thereby, this definition anticipated subsequent trends in research, prevention, and medicine to additionally consider social and mental aspects [2]. In the past, research into well-being has mainly focused on negative attitudes and affect. In particular stress, depression, and anger were identified as independent risk factors for mortality [3], cancer [4], type 2 diabetes mellitus [5], and cardiovascular diseases [6]. However, positive attitudes and affect do represent more than just the opposite of the negative affect scale by having specific and independent health-promoting effects [7–9]. Thus, there is a need for research specifically focused on the impact of well-being as well as positive personality traits and attitudes on health.

Previously, it was shown that a high level of subjective well-being is associated with a decreased mortality by an estimated 7.5 to 10 years increase in life expectancy [10–12]. These findings illustrate the relevance of positive attitudes for individuals, society, as well as health politics, and interestingly the effect size for the association with hard clinical endpoints is comparable to that of established risk factors such as smoking [13]. Several implications of subjective well-being and positive affect on health and potential physiological pathways are discussed in the literature [7–9], suggesting a potential role in chronic disease development. Previous studies investigated a spate of different constructs of subjective well-being ranging from attitudes like optimism and emotional vitality to happiness or having a sense of purpose in life. This complicates the interpretation and the quantification of the effect on chronic disease development and could be a reason for observed conflicting results—for example concerning cancer [14,15] or coronary heart disease in women [16,17].

Life satisfaction represents *“a global assessment of a person’s quality of life according to his/her [the respondent’s] chosen criteria”* [18]. It is well accepted that life satisfaction demonstrates—in contrast to temporary variable positive and negative emotions—the relatively stable component of subjective well-being and refers to cognitive judgments based on a selected set of constantly accessible and stable sources [19,20]. In doing so, the unidimensional construct of life satisfaction integrates important components of life domains such as marriage, health, work, and leisure by individual weightings and rankings, and thus, positive as well as negative aspects [21]. Therefore, life satisfaction is a promising approach to estimate positive psychological attributes in a more comprehensive and convenient way. For these reasons, life satisfaction seems to be an appropriate measure to investigate associations with long-term outcomes in large longitudinal studies, especially since it has been shown that it is quite constant even over a longer period of time [7,9]. Relationships between life satisfaction and mortality [22–24], subsequent work disability [25], and health behavior [8,9,26,27] have already been reported.

Furthermore, previous studies on psychosocial determinants and health recurrently emphasized differences in the extent of examined associations in men and women, wherefore a general gender-specific approach seems to be obvious. In this regard, an underlying net of structural, psychosocial, and behavioral factors, growing out of the social context that is substantially determined by gender roles and connected to specific coping strategies, have been discussed to mediate the differential vulnerability concerning psychosocial conditions [28,29]. Sex-specific differences in disease development pathways due to biological sources such as hormonal and genetic disparities were assumed to play an additional role.

Hence, the aim of our study was to systematically investigate the sex-specific association between life satisfaction and important chronic disease endpoints such as type 2 diabetes mellitus, myocardial infarction, stroke, and cancer in a large prospective study population. 

## Methods

### Ethics statement

All participants of EPIC-Potsdam and EPIC-Heidelberg gave written informed consent and the study was approved by the ethics committees of the Heidelberg Medical Faculty and the Medical Association of the State of Brandenburg.

### Study Population

The European Prospective Investigation into Cancer and Nutrition (EPIC) study is a large multi-centre prospective cohort study that intends to investigate the associations between diet, lifestyle and chronic disease risk with a special focus on cancer. It contains about 520,000 participants in 10 European countries [30,31]. The study population of the present analysis comprised the two German cohorts located in Potsdam and Heidelberg. Participants were randomly selected from the general population of the two cities and their surrounding communities by using residents’ registration offices. A total of 53,088 participants (22,833 men and 30,255 women) aged mainly between 35 and 65 (0.005% outlying this range) were recruited from 1994 to 1998 [32]. They signed a written informed consent before they took part in the baseline examination. Baseline instruments to collect several aspects on diet and lifestyle included self-administered questionnaires, PC-guided interviews, and physical examinations [33]. Participants were excluded from this analysis if they had missing information on life satisfaction (n = 47; 0.1%), covariates (n = 1,082; 2%), diagnosis (n = 15; 0.03%), death (n = 3; 0.005%), or did not participate in the follow-up (n = 1,583; 3%). Altogether, data from 50,358 participants were available for analysis. Prior to analysis, we further excluded endpoint specific prevalent cases, which resulted in a final sample size of 48,411 participants for cancer, 48,075 for type 2 diabetes mellitus, and 48,976 participants for the cardiovascular endpoints stroke and myocardial infarction.

### Measurement of life satisfaction and covariates

At baseline, a PC-guided face-to-face interview that asked for individual characteristics concerning health and lifestyle additionally contained a one-item question to measure global life satisfaction: “What do you think—all in all—how satisfied you are with your life?” The participants were able to choose between the following answers: “very satisfied”, “rather satisfied”, “rather unsatisfied”, “very unsatisfied”, “I don’t know”, and “not specified”. Rather and very unsatisfied participants were merged to “unsatisfied” since only 678 persons (1.4%) reported to be very unsatisfied with their life. Participants who chose “I don’t know” or “not specified” in the life satisfaction question were treated as missing and therefore deleted from the analysis (n = 47; 0.1%). The interview further included questions on smoking behavior (never, former, current) and physical activity (active, moderately active, moderately inactive, inactive) [33]. Categories of physical activity were defined using the Cambridge Index [34]. Consumption of fruits and vegetables (g/day), red meat (g/day), whole-grain bread (g/day), and alcohol (g/day), education (none, primary school, technical school, secondary school, higher education/university), occupation (unemployed/housewife/house husband/maternity leave, retired, part time, full time), and family status (single, married, divorced, widowed) were collected with the help of self-administered questionnaires. Waist-to-hip-ratio (WHR), body mass index (BMI), and blood pressure was measured by trained study staff. Prevalent hypertension was classified by WHO criteria [35].

### Ascertainment of chronic disease incidence

The ascertainment of incident cases of type 2 diabetes mellitus, myocardial infarction, stroke, and cancer included the fourth round of follow-up at Potsdam (2004-2008) and the third round of follow-up at Heidelberg (2004-2006). Diseases were at first ascertained by participants self-report on the disease occurrence, by disease-relevant medication, by changes in diet, or by cause of death information. To secure each incident disease, all diagnoses were further verified by contacting the treating physician and/or clinic to obtain medical records or via linkage to cancer and hospital registries [36]. The diseases were coded in accordance with the *International Statistical Classification of Diseases, 10* ^*th*^ *Revision* (ICD-10) criteria: E11 for type 2 diabetes mellitus, I21 for myocardial infarction, I60, I61, I63, I64 for stroke, and C00-C97 for cancer (except C44: non-melanoma skin cancer).

### Statistical analysis

Descriptive statistics were performed for both sexes combined as well as for men and women separately. Associations between life satisfaction and chronic diseases were investigated by discrete analyses conducted for the endpoints cancer, type 2 diabetes mellitus, myocardial infarction, and stroke. To examine associations between life satisfaction and incidence of chronic diseases, Cox proportional hazards regressions were used systematically with different adjustment models. Life satisfaction was included in three categories whereby people who reported to be very satisfied were selected as the reference group to take into account the potential heterogeneity of the “unsatisfied group”. Time at risk was defined as the time span between the age at study recruitment and the age at diagnosis of the respective disease or the last follow-up (censoring). Models were stratified by integers of age at recruitment and by study center to reduce sensitivity to any respective effects due to varying incidence rates (using the “strata” statement in SAS). This method allows calculating a combined hazard ratio over all strata since different baseline hazard functions but a consistent effect of life satisfaction on disease risk across strata were expected. Supporting this, violations of the Cox proportional hazards assumption for life satisfaction in relation to each disease endpoint were checked and satisfied [37]. In this way, model 1 considered age and study center by stratification. Model 2 was additionally adjusted for established risk factors such as smoking (never, former, current), alcohol intake (≤10 g/day, >10-40 g/day, >40 g/day), physical activity (active, moderately active, moderately inactive, inactive), education (none, primary school, technical school, secondary school, higher education/university), WHR, consumption of fruits & vegetables (g/day), red meat (g/day), and whole-grain bread (g/day). Model 3 was further adjusted for prevalent co-morbidities which may increase the risk of the respective endpoint (cancer: none; type 2 diabetes mellitus: hypertension (yes/no); myocardial infarction and stroke: type 2 diabetes mellitus (yes/no), hypertension (yes/no)). Disease-specific hazard ratios (HR) and approximate 95% confidence intervals (CI) were calculated. Regressions were repeated after exclusion of incident cases within the first 2 years of follow-up to account for potential influences of reverse causality due to modified life satisfaction as consequence of preexisting diseases. To test for gender-specific differences, multiplicative interaction terms relating to life satisfaction categories (sex * life satisfaction category_(i)_) were introduced in the disease-specific fully adjusted models. Within a sensitivity analysis, further interactions were tested between life satisfaction and the considered covariates age (years), alcohol intake (g/day), smoking (categorical), physical activity (categorical), education (categorical), and BMI (kg/m^2^) separately. For that purpose, regarding each possible interaction and endpoint, multiplicative interaction terms were included in the fully adjusted models and then were compared to the respective non-extended models using likelihood ratio tests. If an interaction was detected, a stratified analysis was performed.

The level of statistical significance was set at *p*<0.05 for two-sided testing. All analyses were performed with SAS statistical software (release 9.2; SAS Institute Inc, Cary, North Carolina).

## Results

### Baseline characteristics

The study population consisted of 50,358 participants (57% women). Baseline characteristics are shown for both sexes combined since differences between men and women were only detected with regard to education and BMI (table 1). It’s noteworthy that only 39% of the very satisfied but 65% of the unsatisfied participants belonged to the Potsdam cohort. Participants reporting to be unsatisfied compared to those being very satisfied were slightly younger, less physically active, more frequent smokers, less likely to be married, more likely to be unemployed, and showed an increased consumption of red meat and total energy. Furthermore, the unsatisfied group contained a higher percentage of people with prevalent type 2 diabetes mellitus and hypertension. Unsatisfied men were on average higher educated and unsatisfied women tended to have a higher BMI (data not shown). A clear interaction between sex and life satisfaction concerning cancer risk (p-value of likelihood ratio test for interaction = 0.02) was revealed. Therefore, the intended sex-specific approach of the following analyses was confirmed and the following analyses were performed separately for men and women. Type 2 diabetes mellitus, stroke, and myocardial infarction showed no interaction with sex (p-values of likelihood ratio tests for interaction were 0.15, 0.64, and 0.26, respectively).

**Table 1:** Means (standard deviation) and proportions of baseline characteristics according to life satisfaction categories within EPIC Germany.

	**Life satisfaction**		
	**Very satisfied**	**Rather satisfied**	**Unsatisfied**
**n**	15,838	29,513	5,007
**Study center** (% Potsdam)	39	57	65
**Age** (years)	50.8 (8.6)	50.0 (8.6)	49.7 (8.4)
**BMI** (kg/m^2^)	26.1 (4.1)	26.2 (4.3)	26.5 (4.8)
**WHR**	0.860 (0.098)	0.859 (0.098)	0.864 (0.098)
**Physical activity** (%)			
Inactive	14	17	25
Moderately inactive	36	37	36
Moderately active	27	26	23
Active	23	20	16
**Smoking** (%)			
Nonsmoker	46	46	42
Former smoker	34	33	30
Current smoker	21	22	28
**Highest school level** (%)			
None	1	1	1
Primary school completed	24	23	22
Technical/professional school	36	36	36
Secondary school	7	7	7
University	33	36	35
**Family status** (%)			
Single	6	9	11
Married	83	76	67
Divorced	8	11	17
Widowed	3	4	5
**Prevalent myocardial infarction** (%)	2	2	3
**Prevalent stroke** (%)	1	1	2
**Prevalent cancer** (%)	4	4	5
**Prevalent type 2 diabetes mellitus** (%)	4	5	7
**Prevalent hypertension** (%)	35	41	46
**Total energy intake** (kJ/day)	8,374 (2,926)	8,620 (2,978)	8,837 (3,407)
**Vegetables & fruits** (g/day)	244 (139)	242 (124)	240 (128)
**Whole grain bread** (g/day)	48 (55)	46 (55)	44 (57)
**Red meat** (g/day)	92 (64)	98 (65)	101 (71)
**Alcohol consumption** (g/day)	16.9 (21.7)	15.5 (20.3)	15.8 (22.8)
**Occupation** (%)			
Unemployed / housewife / house husband / maternity leave	12	12	22
Retired	16	16	16
Part time	18	15	14
Full time	54	58	47

### Life Satisfaction and Cancer Risk

After an average of 8 years of follow-up, cancer was diagnosed in 1,223 men and 1,070 women. In men, there was no association between life satisfaction and cancer risk (table 2). However, an interaction between life satisfaction and alcohol was observed (p-value of likelihood ratio test for interaction = 0.001) in men. Stratified analysis showed that low life satisfaction was associated with an increased cancer risk in men consuming less than 10g alcohol per day (HR for linear trend: 1.19; 95% CI: 1.01-1.41) and a contrary—but only borderline significant—association was observed in men consuming more than 40g alcohol per day (HR for linear trend: 0.82; 95% CI: 0.67-1.01).

In contrast, “unsatisfied” compared to “very satisfied” women showed a 45% increase in cancer risk even after adjusting for established risk factors in model 2 (HR: 1.45; 95% CI: 1.18-1.78). In “rather satisfied” women no significant change in risk was observed but yet a negative linear trend across all categories of life satisfaction (p for trend = 0.005). After excluding incident cases within the first 2 years of follow-up, the association between life satisfaction and cancer incidence in women remained robust.

**Table 2:** Sex-specific and multivariable-adjusted hazard ratios (HR) and 95%-confidence intervals (CI) of cancer incidence according to life satisfaction within EPIC Germany

Cancer				
	Life satisfaction			
	Very satisfied	Rather satisfied	Unsatisfied	
**Men**	428 cases / n = 6,646	692 cases / n = 12,383	103 cases / n = 2,061	
	(ref.)	**HR (95%-CI)**	**HR (95%-CI)**	***P*-trend**
Model 1	1	1.05 (0.93-1.18)	1.03 (0.83-1.29)	0.38
Model 2	1	1.03 (0.91-1.17)	1.00 (0.81-1.25)	0.76
**Women**	337 cases / n = 8,594	601 cases / n = 16,011	132 cases / n = 2,716	
	(ref.)	**HR (95%-CI)**	**HR (95%-CI)**	***P*-trend**
Model 1	1	1.05 (0.92-1.20)	1.47 (1.20-1.81)	0.08
Model 2	1	1.04 (0.91-1.19)	1.45 (1.18-1.78)	0.005

**Model 1**: Cox proportional hazards regression stratified by age and study center

**Model 2**: model 1 with additional adjustment for smoking (never, former, current), alcohol intake (≤10 g/day, >10-40 g/day, >40 g/day), physical activity (active, moderately active, moderately inactive, inactive), education (none, primary school, technical school, secondary school, higher education/university), WHR, consumption of fruits & vegetables (g/day), red meat (g/day), and whole-grain bread (g/day)

### Life satisfaction and type 2 diabetes mellitus risk

Type 2 diabetes mellitus was diagnosed in 1,112 men and 728 women during an average of 8 years follow-up time. As with cancer there was no association between life satisfaction and diabetes risk in men (table 3). But we found a significant interaction between life satisfaction and smoking (p-value of likelihood ratio test for interaction = 0.016). Stratified analysis suggested that diabetes risk tends to decrease by a decline of life satisfaction in never smokers (HR for linear trend: 0.81; 95% CI: 0.65-1.00). In current and former smokers no significant risk association with life satisfaction was detected.

With respect to women, the age- and center-stratified risk increase in model 1 was 21% (HR: 1.21; 95% CI: 1.02-1.43) for “rather satisfied” and 53% (HR: 1.53; 95% CI: 1.19-1.97) for “unsatisfied” women compared to the “very satisfied”. After additional consideration of established risk factors (model 2) and prevalent hypertension (model 3), there was still a consistent trend for a negative linear relationship between life satisfaction and type 2 diabetes mellitus incidence across categories (p for trend = 0.04 for model 2 and 3). After excluding incident type 2 diabetes mellitus cases within the first 2 years of follow-up, a significant linear association over descending life satisfaction categories was restricted to the model only controlled for age and study center (HR for linear trend: 1.20; 95% CI: 1.05-1.37).

**Table 3:** Sex-specific and multivariable-adjusted hazard ratios (HR) and 95%-confidence intervals (CI) of type 2 diabetes mellitus incidence according to life satisfaction within EPIC Germany.

**Type 2 diabetes mellitus**				
	**Life satisfaction**			
	**Very satisfied**	**Rather satisfied**	**Unsatisfied**	
**Men**	348 cases/n = 6,448	653 cases/n = 11,898	111 cases/n = 1,943	
	(ref.)	**HR (95%-CI)**	**HR (95%-CI)**	***P*-trend**
Model 1	1	1.07 (0.93-1.22)	1.12 (0.90-1.40)	0.07
Model 2	1	1.04 (0.91-1.19)	1.05 (0.84-1.31)	0.55
Model 3	1	1.02 (0.89-1.16)	1.00 (0.80-1.24)	0.93
**Women**	202 cases/n = 8,793	438 cases/n = 16,249	88 cases/n = 2,744	
	(ref.)	**HR (95%-CI)**	**HR (95%-CI)**	***P*-trend**
Model 1	1	**1.21 (1.02-1.43)**	**1.53 (1.19-1.97)**	**0.0004**
Model 2	1	1.17 (0.99-1.39)	1.27 (0.98-1.65)	**0.04**
Model 3	1	1.17 (0.98-1.39)	1.27 (0.98-1.64)	**0.04**

**Model 1**: Cox proportional hazards regression stratified by age and study center

**Model 2**: model 1 with additional adjustment for smoking (never, former, current), alcohol intake (≤10 g/day, >10-40 g/day, >40 g/day), physical activity (active, moderately active, moderately inactive, inactive), education (none, primary school, technical school, secondary school, higher education/university), WHR, consumption of fruits & vegetables (g/day), red meat (g/day), and whole-grain bread (g/day)

**Model 3**: model 2 with additional adjustment for prevalent hypertensionHR: hazard ratios; CI: confidence intervals

### Life satisfaction and cardiovascular disease risk

During a mean follow-up of 8 years, incident stroke occurred in 253 men and 187 women and incident myocardial infarction was diagnosed in 433 men and 129 women. Concerning stroke, men showed a significant association in the age- and center-stratified model 1 at which compared to “very satisfied” men a 37% increased risk in “rather satisfied” men (HR: 1.37; 95% CI: 1.03-1.84) and a 60% increased risk in “unsatisfied men” (HR: 1.60; 95% CI: 1.02-2.49) was detected (table 4). Adjustment for established lifestyle risk factors (model 2) outweighed this elevated risk for stroke but a significant linear relationship across all groups of life satisfaction remained (p for trend = 0.03), which disappeared after further adjustment for prevalent hypertension and type 2 diabetes mellitus (model 3). The observed risk association persisted even if incident cases within the first 2 years of follow-up were excluded. In women, “unsatisfied” participants showed an increased risk of stroke compared to the “very satisfied” (HR: 1.99; 95% CI: 1.24-3.19). A slightly attenuated but still marked 69% increased risk was observed after consideration of common risk factors and prevalent hypertension in model 3 (HR: 1.69; 95% CI: 1.05-2.73). Excluding incident cases within the first 2 years of follow-up, the clear linear risk increase over descending life satisfaction categories in women could only be confirmed in the only age- and center-controlled model 1 and became borderline significant under consideration of further risk factors (HR for linear trend: 1.37; 95% CI: 1.05-1.79 for model 1, HR for linear trend: 1.28; 95% CI: 0.99-1.67 for model 2, and HR for linear trend: 1.27; 95% CI: 0.98-1.66 for model 3).

In contrast, no significant association between life satisfaction and risk of myocardial infarction was detected except for a borderline significant trend across categories in the age- and center-stratified model in women (p for trend = 0.052, table 5).

**Table 4:** Sex-specific and multivariable-adjusted hazard ratios (HR) and 95%-confidence intervals (CI) of stroke incidence according to life satisfaction within EPIC Germany.

**Stroke**				
	**Life satisfaction**			
	**Very satisfied**	**Rather satisfied**	**Unsatisfied**	
**Men**	68 cases/n = 6,545	156 cases/n = 12,135	29 cases/n = 1,986	
	(ref.)	**HR (95%-CI)**	**HR (95%-CI)**	***P*-trend**
Model 1	1	**1.37 (1.03-1.84)**	**1.60 (1.02-2.49)**	**0.02**
Model 2	1	1.33 (0.998-1.78)	1.54 (0.98-2.40)	**0.03**
Model 3	1	1.30 (0.97-1.74)	1.40 (0.89-2.19)	0.07
**Women**	49 cases/n = 8,927	110 cases/n = 16,570	28 cases/n = 2,813	
	(ref.)	**HR (95%-CI)**	**HR (95%-CI)**	***P*-trend**
Model 1	1	1.25 (0.89-1.76)	**1.99 (1.24-3.19)**	**0.01**
Model 2	1	1.22 (0.86-1.71)	**1.80 (1.12-2.89)**	**0.02**
Model 3	1	1.20 (0.85-1.69)	**1.69 (1.05-2.73)**	**0.04**

**Model 1**: Cox proportional hazards regression stratified by age and study center

**Model 2**: model 1 with additional adjustment for smoking (never, former, current), alcohol intake (≤10 g/day, >10-40 g/day, >40 g/day), physical activity (active, moderately active, moderately inactive, inactive), education (none, primary school, technical school, secondary school, higher education/university), WHR, consumption of fruits & vegetables (g/day), red meat (g/day), and whole-grain bread (g/day)

**Model 3**: model 2 with additional adjustment for prevalent hypertension and type 2 diabetes mellitus

**Table 5:** Sex-specific and multivariable-adjusted hazard ratios (HR) and 95%-confidence intervals (CI) of myocardial infarction incidence according to life satisfaction within EPIC Germany.

**Myocardial infarction**				
	**Life satisfaction**			
	**Very satisfied**	**Rather satisfied**	**Unsatisfied**	
**Men**	142 cases/n = 6,545	241 cases/n = 12,135	50 cases/n = 1,986	
	(ref.)	**HR (95%-CI)**	**HR (95%-CI)**	***P*-trend**
Model 1	1	1.02 (0.82-1.26)	1.36 (0.98-1.90)	0.17
Model 2	1	0.98 (0.79-1.21)	1.27 (0.91-1.77)	0.36
Model 3	1	0.97 (0.78-1.20)	1.13 (0.81-1.58)	0.69
**Women**	32 cases/n = 8,927	82 cases/n = 16,570	15 cases/n = 2,813	
	(ref.)	**HR (95%-CI)**	**HR (95%-CI)**	***P*-trend**
Model 1	1	1.49 (0.98-2.26)	1.66 (0.89-3.09)	0.052
Model 2	1	1.38 (0.91-2.10)	1.41 (0.75-2.63)	0.16
Model 3	1	1.39 (0.92-2.12)	1.26 (0.67-2.38)	0.26

**Model 1**: Cox proportional hazards regression stratified by age and study center

**Model 2**: model 1 with additional adjustment for smoking (never, former, current), alcohol intake (≤10 g/day, >10-40 g/day, >40 g/day), physical activity (active, moderately active, moderately inactive, inactive), education (none, primary school, technical school, secondary school, higher education/university), WHR, consumption of fruits & vegetables (g/day), red meat (g/day), and whole-grain bread (g/day)

**Model 3**: model 2 with additional adjustment for prevalent hypertension and type 2 diabetes mellitus

## Discussion

The present study showed negative associations between life satisfaction and the development of chronic diseases with clear gender differences that were independent of established risk factors. Cancer and stroke incidence were increased in women with lower levels of life satisfaction. Also in women, life satisfaction showed an inverse relationship by trend for type 2 diabetes mellitus; however, no association with myocardial infarction was seen. After excluding the first two years of follow-up to avoid potential reverse causality, cancer risk remained robust. Regarding stroke and type 2 diabetes mellitus, at least a partial influence of an incipient disease on life satisfaction measurement could not be excluded. In men, life satisfaction only showed a negative association with stroke incidence but not independent from common risk factors. These findings confirmed an association between life satisfaction and chronic disease incidence in women and add to the still limited and partly controversial body of evidence regarding the role of positive attitudes and personality traits on disease incidence.

The results of previous studies concerning psychological factors and cancer incidence are rather inconsistent, and many studies specifically focused on breast cancer [4,14,15,38,39]. Concerning positive attitudes, happiness, living a life worth living, and optimism were reported to be negatively associated with breast cancer [40,41]. However, some of these associations may rest on study design and insufficient consideration of possible confounding factors. Although an inverse association between life satisfaction and breast cancer risk could not be approved so far [42], it cannot be excluded that our results concerning cancer incidence in women could at least partly been driven by this association. In fact, breast cancer is the most common cancer in women with a proportion of 28% in total female cancer incidence [43]. The negative association between life satisfaction and cancer risk may also be related to the stress buffering effect of a high life satisfaction [4,9]. Studies suggested an influence of psychosocial factors—particularly stress—on virus-related immunogenetic cancer incidence [4,44]. However, the percentage of infection-attributable cancers in developed countries amounts to only 7.7% of all cancer cases in both sexes [45]. Thus, this fact is unlikely to explain the differences in men and women. More detailed analysis according different types of cancer could provide a deeper insight to explain the observed sex differences, but would require a larger sample size.

Regarding the endpoint type 2 diabetes mellitus, a negative association with life satisfaction was found for women in all three models by trend. After deleting incident cases within the first 2 years of follow-up, a significant risk increase could only be seen in the model only stratified by age and study center without consideration of further established risk factors. In fact, it is commonly accepted that type 2 diabetes mellitus development is strongly affected by the considered lifestyle and clinical risk factors wherefore the relation between life satisfaction and diabetes could be mediated by them. The observed weak association between life satisfaction and type 2 diabetes mellitus risk in women may indirectly confirm previous studies which found negative affect and experiences such as stress, depressive symptoms, anger, hostility, sleeping problems, and anxiety to be associated with type 2 diabetes mellitus incidence [5]. Life satisfaction might attenuate the influence of negative affect. However, the connection between positive affect and type 2 diabetes mellitus risk is only rarely investigated so that our results could provide a first basis.

In our study, stroke appeared to be the only endpoint associated with life satisfaction in men as well as in women, although the association was not independent from established risk factors and prevalent diseases. Stroke risk is known to be strongly impacted by an unhealthy lifestyle and prevalent hypertension as well as type 2 diabetes mellitus. Therefore, the influence of life satisfaction on stroke risk could be mediated by these factors. Our results corroborate findings on happiness or positive affect and stroke risk, even though they were mainly detected in men [17,46]. Risk associations between positive affect and coronary heart disease, which were previously reported for positive emotion, optimism, having a sense of purpose in life, and emotional vitality [16,47–51], could not be confirmed in our study. The low number of cases in the unsatisfied group—particularly in women—might provide a possible explanation for the missing association in our analyses. However, one study that investigated positive affect and coronary heart disease risk failed to approve a relationship as well [52].

Our results suggest a stronger relationship between life satisfaction and chronic disease incidence in women than in men. This is in line with previous findings indicating that both structural determinants (socio-economic, age, social support, family arrangement) and psychosocial factors (critical life events, stress, psychological resources) are generally more important for women’s health including self-rated health, functional health, distress, and chronic illness [28]. In contrast, a large meta-analysis on well-being and health declared that the impact seems to be more explicit in men [7]. However, this assumption was mainly based on longevity and corroborates studies which showed a stronger negative association between life satisfaction or happiness and mortality in men [23,53,54]. Indeed, further studies on psychosocial factors and health outcomes like heart rate, stroke incidence, and coronary heart disease indicated similar risk increase in men [17,46,47,55]. But these results might also be influenced by the chosen construct of well-being, cultural differences, or the investigated outcome. For this reason, they are not directly comparable to the present findings. A possible bias due to over-euphemistic answers in men in the face-to-face interview with which life satisfaction was measured is unlikely because men and women showed a quite similar distribution over the three different categories of life satisfaction. Another reason could be that women tend to consult their doctor or make health check-ups more frequently than men [56,57] which might have influenced the date of diagnosis and thereby the risk associations. On physiological level, the observed sex differences may be due to genetic disparities and steroid hormones that influences well-known risk factors such as body fat distribution, or may directly affect type 2 diabetes mellitus incidence by regulating insulin resistance [58]. They may also play a role in manifestation and symptoms of particularly coronary heart diseases [59,60], as well as susceptibility and location of cancer [61]. Hence, it is imaginable, that these sex differences in disease development pathways also offer different possibilities of being impacted by psychosocial influences.

Unsatisfied participants showed a less healthy lifestyle and as a result a larger amount of common risk factors for the investigated endpoints, which was also reported by previous studies [8,9,26,27]. Furthermore, this relationship seems to be bidirectional which means that life satisfaction influences lifestyle and vice versa [26]. The association between life satisfaction and disease could be mediated by lifestyle factors. For this reason, we considered common disease-related covariates in the analysis. The results showed an association between life satisfaction and cancer in women which was independent of considered covariates. In contrast, the relationship between life satisfaction and stroke in both sexes as well as type 2 diabetes mellitus in women did not persist independently after controlling for common risk factors. This underlines the well-known importance of lifestyle regarding the development of these chronic diseases. Because of the bidirectional relationship between life satisfaction and lifestyle [26], the quantification of the effect in absolute terms remains difficult, even after excluding the lifestyle dominated pathway in the statistical models. Therefore, it can be assumed that the real effect size—which additionally includes the life satisfaction impact mediated by lifestyle factors—is possibly stronger and the results have to be interpreted with caution regarding the statistical models used.

To clarify the biological plausibility of the effect of life satisfaction on chronic disease development, two main models are discussed in the literature. In fact, the two models may complement each other. The first so-called *Main-*(*Direct*)*-Effect-Model* describes the influence of positive affect on behavior and physiological mechanisms [9]. Thus, a higher level of positive affect and attitudes may promote a healthier lifestyle, social support, immune function, and the endogenous opioid system [8,9,26,27]. In addition, the activation of the autonomic nervous system may decrease heart rate, blood pressure, and levels of the stress-related hormones epinephrine and norepinephrine [9]. Furthermore, a diminished release of cortisol, which plays an important role in many physiological outcomes such as immune and inflammatory diseases, as well as an increased release of oxytocin and growth hormone is caused by a hypothalamic–pituitary–adrenal axis activation [9]. The *Stress-Buffering-Model* assumes that positive affect and its related social environment promote the development of important social, psychological, and physiological resources to counteract stress in its intensity, duration, and pathogenic effects on the body [9]. Observed differences in the strengths of association between life satisfaction and disease endpoints may be due to the different importance of stress-related factors in disease-specific pathogenesis.

The present study has a number of important strengths including the size of the study population, the prospective cohort design, the gender-specific analysis, and the separate examination of different endpoints. Potential limitations include the face-to-face interview to measure life satisfaction and the limited number of possible answers what may decrease sensitivity and mask real associations. On the other hand, the very large study population should provide adequate sensitivity and the limited answer possibilities increase reliability as well as it helps to avoid irrelevant results [62]. Temporal stability of life satisfaction might be another constraint because it was measured only once at baseline and the study design assumes a constant exposition during follow-up. Nevertheless, previous studies showed an acceptable stability with only a small proportion of people changing their satisfaction level over time [63,64]. Finally, the study population is not representative of the general population as it consists of a higher percentage of health-conscious and graduated participants [32]. Interestingly, we observed different distributions of life satisfaction between the two German cities included in the cohort. This could be due to the societal upheavals in the years before the study’s baseline which may have primarily affected Potsdam located in the former German Democratic Republic. However, the association between life satisfaction and disease incidence seems not to be influenced since no interaction with study center was detected. Finally, negative affect was not measured directly in this study and could therefore not be considered in the statistical models. Nevertheless, the construct of life satisfaction is based on a global cognitive assessment of life and therefore represents an integration of both, positive as well as negative aspects.

In conclusion, life satisfaction was negatively associated with risk of cancer, stroke, and type 2 diabetes mellitus, particularly in women. The relation to cancer incidence was additionally independent of common risk factors. There is some evidence that the effect of poor life satisfaction could additionally be mediated by a disadvantageous lifestyle. The present study is among the first that investigated the association between life satisfaction and incidence of major chronic diseases. Because the considered disease endpoints are the most frequent and cost-intensive diseases in our society, life satisfaction and psychosocial factors deserve closer attention, particular under consideration of sex differences. Further studies are needed on potential prevention and intervention strategies both on the individual and the societal level.
